# Energy performance of compressed biomethane gas production from co-digestion of *Salix* and dairy manure: factoring differences between *Salix* varieties

**DOI:** 10.1186/s13068-023-02412-1

**Published:** 2023-11-03

**Authors:** Saurav Kalita, Jonas A. Ohlsson, Hanna Karlsson Potter, Åke Nordberg, Mats Sandgren, Per-Anders Hansson

**Affiliations:** 1 Department of Energy and Technology, Swedish University of Agricultural Sciences, P.O. Box 7032, 750 07 Uppsala, Sweden; 2 Department of Molecular Sciences, Swedish University of Agricultural Sciences, P.O. Box 7015, 750 07 Uppsala, Sweden

**Keywords:** *Salix*, Energy analysis, Biogas, Lignocellulosic biomass, Short-rotation coppice willow, Systems perspective, Biomethane, Energy balance

## Abstract

Biogas from anaerobic digestion is a versatile energy carrier that can be upgraded to compressed biomethane gas (CBG) as a renewable and sustainable alternative to natural gas. Organic residues and energy crops are predicted to be major sources of bioenergy production in the future. Pre-treatment can reduce the recalcitrance of lignocellulosic energy crops such as *Salix* to anaerobic digestion, making it a potential biogas feedstock. This lignocellulosic material can be co-digested with animal manure, which has the complementary effect of increasing volumetric biogas yield. *Salix* varieties exhibit variations in yield, composition and biomethane potential values, which can have a significant effect on the overall biogas production system. This study assessed the impact of *Salix* varietal differences on the overall mass and energy balance of a co-digestion system using steam pre-treated *Salix* biomass and dairy manure (DaM) to produce CBG as the final product. Six commercial *Salix* varieties cultivated under unfertilised and fertilised conditions were compared. Energy and mass flows along this total process chain, comprising *Salix* cultivation, steam pre-treatment, biogas production and biogas upgrading to CBG, were evaluated. Two scenarios were considered: a base scenario without heat recovery and a scenario with heat recovery. The results showed that *Salix* variety had a significant effect on energy output–input ratio (R), with R values in the base scenario of 1.57–1.88 and in the heat recovery scenario of 2.36–2.94. In both scenarios, unfertilised var. Tordis was the best energy performer, while the fertilised var. Jorr was the worst. Based on this energy performance, *Salix* could be a feasible feedstock for co-digestion with DaM, although its R value was at the lower end of the range reported previously for energy crops.

**Supplementary Information:**

The online version contains supplementary material available at 10.1186/s13068-023-02412-1.

## Introduction

Fossil fuels are the major source of primary energy across the world [1] and are also the main source of anthropogenic emissions of greenhouse gases (GHGs) leading to global warming [2]. To limit global warming to 1.5 °C, global GHG emissions need to peak before 2025, be reduced by 43% by 2030, and reach net zero by the early 2050s, according to the latest IPCC assessments [3]. Countries, regions, cities and companies representing 85% of the world’s population and 90% of GDP (PPP) have set net zero targets or have pledged to limit global warming within this century [4]. Another problem with fossil fuels is the unequal distribution of reserves, leading to inequalities in supply and demand and dependence on producing nations. This leads to energy insecurity, geo-political issues and conflicts.

Sustainable bioenergy is an important part of fossil-fuel free energy production and energy security efforts, by providing viable replacements for solid, liquid and gaseous fossil fuels. Bioenergy can be particularly important in sectors where fossil fuels are difficult to replace (e.g. heavy industry, aviation, heavy transportation). In pathways to reach net-zero emissions by 2050, bioenergy supply is predicted to grow from 65 EJ in 2020 to 100–248 EJ by 2050 [3, 5]. Biogas produced by anaerobic digestion of organic matter can be used for heat and power production and can be upgraded to biomethane by removing CO_2_ and trace gases. It can use existing gas infrastructure and technologies, such as pipelines [6] and natural gas engines. Biomethane can be compressed in a similar way to natural gas to make compressed biomethane gas (CBG), as an alternative to compressed natural gas. This makes biomethane an attractive fossil-free vehicle fuel option. In the IEA net zero emissions scenario [5], biogas use reaches 14 EJ in 2050, from 2.1 EJ in 2020. The REPowerEU action plan envisions boosting biomethane production to 35 bcm by 2030 to reduce dependence on Russian natural gas [7]. Therefore, there is interest in increasing biogas production in a sustainable manner to reduce natural gas use.

Different feedstocks are being investigated to meet the growing demand for bioenergy and realize its potential. Energy crops have played an important role in increasing biogas production in some countries such as Germany [8], but use of conventional energy crops such as sugar beet and maize can lead to conflicts with food production and supply. Therefore, there is a need for alternative feedstocks, such as waste streams, short-rotation lignocellulosic crops and feedstocks, that can be cultivated on non-agricultural land and which are not used for food and feed. In both the EU [9] and Sweden [10, 11], organic residues and energy crops offer the greatest potential for increasing biogas production. According to the IEA roadmap for net zero emissions by 2050 [5], organic waste streams and short-rotation woody crops will be the main sources of the future global bioenergy supply.

Animal manure has great potential for biogas production, with the added benefit of avoiding atmospheric methane emissions from manure decomposition [12], which makes it an attractive option for meeting climate targets. However, manure usually has a very high moisture content, leading to low organic loading rate (OLR), resulting in low volumetric biogas production. A co-digestion system to supplement manure with another feedstock, such as lignocellulosic material, can achieve an increase in volumetric biogas yield without compromising hydraulic retention time (HRT) [13].

Lignocellulosic biomass is a very abundant type of biomass and is relatively economical to produce, but typically has higher recalcitrance than other biomass sources [14]. Recalcitrance can be defined as the resistance of the biomass to release of sugars for fermentation or degradation, which is the major barrier to their conversion to biofuels [15]. Pre-treatment methods can help reduce recalcitrance in lignocellulosic biomass by increasing the accessibility of holocellulose (cellulose and hemicellulose) to microorganisms, improving both the rate and yield of biogas production [16].

Potential sources of sustainable and renewable lignocellulosic biomass include short-rotation coppice systems such as *Salix* plantations. *Salix* plantations have the benefits of relatively short growth cycles of 2–5 years, multiple harvests from the same plantation for 20–25 years, vegetative propagation, simple management practices and high net energy return. They can also provide the additional benefits of soil carbon sequestration, phytoremediation, acting as flood barriers and windbreaks, increased biodiversity and pollinator attraction. *Salix* biomass is thus a promising feedstock for biogas production systems, where it can be co-digested with other substrates such as animal manure [17, 18].

In recent decades, breeding programmes have developed several newer varieties of *Salix*. Studies show that there are significant differences between these *Salix* varieties in terms of biomass yield [19], biomass quality [20, 21], physiological and morphological traits [22, 23], biomethane potential (BMP) [24], soil ecology and response to fertilisation [25]. It is common practice to assume average characteristics for energy crops such as *Salix* in systems studies. There is a lack of analyses that consider varietal differences when examining the energy and mass flow of biogas production systems. These differences should be taken into account when exploring the potential of *Salix*-based biogas production systems, as they can have a significant influence on system parameters and performance.

This study analyzed a biomethane production system using co-digestion of pre-treated *Salix* biomass with dairy manure. The aims were to evaluate energy and mass flows along the total process chain for six selected *Salix* varieties, cultivated under fertilised and unfertilised conditions, and to compare the energy performance of the varieties. A broad cradle-to-grave scope was applied in the analysis starting with *Salix* cultivation and ending with final production of CBG and digestate application to soil.

## Materials and methods

### System boundaries and description

**Fig. 1:**
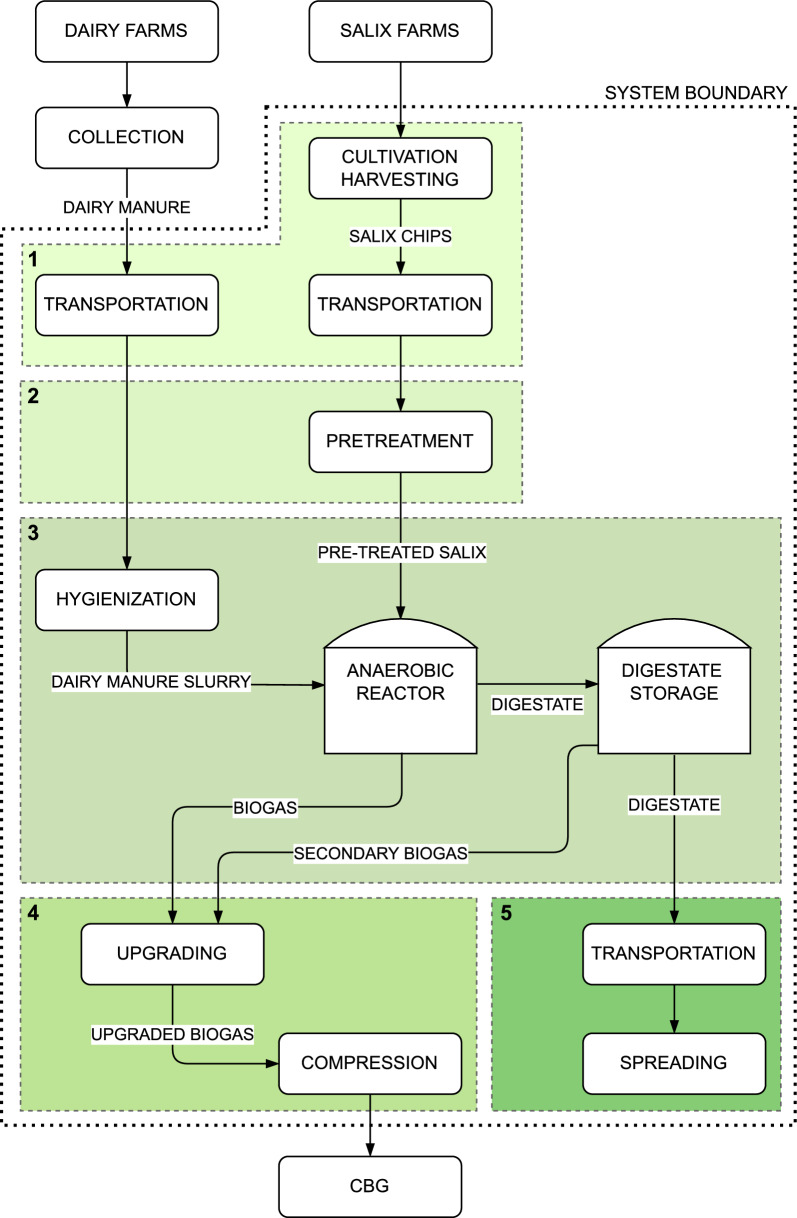
System boundaries of the compressed biomethane gas (CBG) production system analysed in this study

A life-cycle perspective was used to identify and determine the mass and energy flows in a biomethane production system involving co-digestion of steam pre-treated *Salix* biomass and dairy manure (DaM) to produce CBG as a final product. The analysis considered a Swedish context, with the study region assumed to be in Uppsala, central Sweden. The system was assumed to handle a feeding rate of 300 kg/h of dry *Salix* biomass, with all other flows calculated based on this parameter. The system boundaries used in the analysis are shown in Fig. 1, where arrows indicate the material flows within the different sub-systems. The system was divided into five stages:

1. Stage 1 (Raw materials): Cultivation and harvest of the different *Salix* varieties and transportation to the biogas plant. Transportation of DaM from farms to the biogas plant was also included, while its production was excluded from the system.
2. Stage 2 (*Salix* pre-treatment stage): Pre-treatment of *Salix* by SO_2_-catalysed steam explosion of *Salix* at 185 °C for 4 min.
3. Stage 3 (Biogas production): Hygienisation of DaM at 70 °C before adding it to the pre-treated *Salix* for co-digestion in an anaerobic digester at 37 °C. DaM and *Salix* substrates were mixed in a 1:1 volatile solids (VS) ratio with 10% TS content for feeding the anaerobic digester, and a HRT of 45 days was assumed. The biogas produced progressed to stage 4 for upgrading, while the digestate was directed to a storage tank under ambient conditions. The digestate was assumed to be stored in the tank for 30 days, during which further degradation occurred, leading to secondary production of biogas. This secondary biogas was added to the primary biogas flow for upgrading.
4. Stage 4 (Upgrading): The raw biogas was upgraded to bio-methane by removing CO_2_ using a wet scrubber and compressed to transport-grade CBG.
5. Stage 5 (Digestate use): The digestate was transported from the storage tank to agricultural fields and spread as a liquid fertiliser.

### Raw materials

#### *Salix* biomass

Use of biomass from six commercial *Salix* varieties grown under fertilized and unfertilized conditions was compared. The varieties were: ‘Björn’ (*Salix schwerinii* E. Wolf. × *S. viminalis* L.), ‘Gudrun’ (*S. burjatica* Nasarow × *S. dasyclados* Wimm.), ‘Jorr’ (*S. viminalis*), ‘Loden’ (*S. dasyclados*), ‘Tora’ (*S. schwerinii* × *S. viminalis*) and ‘Tordis’ (*S. schwerinii* × *S. viminalis*) × *S. viminalis*). *Salix* growth and cultivation data were obtained from a field trial in Uppsala during 2001–2017 [26]. The varieties cultivated under fertilised conditions received 100 kg N, 14 kg P and 47 kg K per ha and year. The suffixes F0 and F + are used hereafter to refer to unfertilised and fertilised conditions, respectively. The plantations were managed in a three-year cutting cycle, with winter harvests.

*Salix* biomass samples were collected in 2019 and chipped with a compost chipper, after which compositional analysis and BMP assays were performed, the details of which are presented in the supplementary material (SM). Compositional analysis was performed on the extractives, carbohydrate and lignin components of the *Salix* samples. For the BMP assay tests, samples were first steam-exploded under process conditions of 185 °C for 4 min with 2% (mass/mass) SO_2_ as a catalyst. A BMP assay was performed on the samples using inoculum from a wastewater treatment plant, with an inoculum-to-substrate ratio of 3:1 on a volatile solids basis. Cellulose and inoculum controls were included in the assay. Full details of sample preparation and BMP test conditions can be found in the SM.

The composition of untreated *Salix* biomass (cellulose and hemicellulose content of the *Salix* varieties) was calculated from analyzed sugar composition after acid hydrolysis for the different varieties (Additional file 1: Table S1). The cellulose content was considered equivalent to the sum of glucose and cellobiose content. Hemicelluloses were considered to be the polysaccharide forms of xylose, arabinose, mannose and galactose in the concentrations reported. The concentration of polymeric sugars was calculated using anhydro correction factors from the corresponding monomeric sugar as described by Sluiter et al. [27]. Lignin was expected to remain unchanged between untreated and steam-treated samples, as lignin generally does not depolymerise under mild steam treatment conditions. The composition of the untreated *Salix* biomasses and their BMP values are presented in Table 1. The composition values were used as inputs for the process modelling.

**Table 1:** Polysaccharide composition, volatile solids (VS) content and biomethane potential (BMP) of the six selected Salix varieties under unfertilised (F0) and fertilised (F+) conditions (adapted from analytical values in Additional file 1: Table S1)

Variety	Lignin (%VS)	Cellulose (%VS)	Hemi-cellulose				VS (%TS)	BMP (mL/gm VS)
			Xylan **(**%VS**)**	Galactan **(**%VS**)**	Arabinan **(**%VS**)**	Mannan **(**%VS**)**		
Björn F0	24.5	54.0	9.4	1.9	0.4	1.8	97.9	194
Björn F+	24.7	51.3	8.9	1.6	0.4	1.6	98.1	232
Gudrun F0	28.0	50.0	9.2	1.6	0.6	1.6	97.7	246
Gudrun F+	28.7	48.8	8.3	1.7	0.6	1.5	97.3	235
Jorr F0	28.1	49.3	8.6	2.4	0.8	2.3	97.7	216
Jorr F+	27.8	46.9	7.8	2.0	0.9	2.2	98.1	190
Loden F0	29.0	47.3	8.4	1.7	0.7	1.8	96.9	236
Loden F+	29.6	47.3	8.5	1.8	0.8	2.0	97.3	251
Tora F0	29.1	48.0	9.4	2.1	0.7	1.9	97.3	246
Tora F+	26.7	46.1	9.0	1.6	0.6	2.1	97.8	248
Tordis F0	26.0	52.3	9.0	1.9	0.5	2.1	98.1	271
Tordis F+	26.2	50.4	8.9	1.6	0.4	1.8	98.2	268

**Fig. 2:**
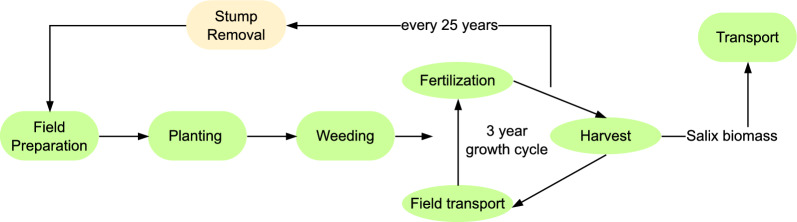
Illustration of the Salix cultivation system analysed

Analysis of *Salix* cultivation and harvest included field preparation, management operations, harvesting and transportation (Fig. 2). Production of fertilisers, pesticides and *Salix* cuttings used as inputs to cultivation was also included. The harvested *Salix* biomass was in the form of chips and was assumed to be transported an average distance of 100 km to the biogas production plant. Energy and material flows for the varieties (Additional file 1: Tables S2–S4) were based on the *Salix* production system covered by Kalita et al. [28]. All agricultural, transport and processing machinery were presumed to use fossil diesel as fuel.

#### Dairy manure

The composition and BMP characteristics of the DaM used as the second feedstock in the co-digestion system (Table 2) were based on averages of DaM data in the literature. Using proportions calculated from reported yields of manure hydrolysis by Chen et al. [29] and Wen et al. [30], the hemicellulose content was divided into arabinose, galactose and xylose. Literature sources [31–35] report BMP values within the range 51–264.3 mL CH_4_/gVS, with an average value of 211.5 ml CH_4_/gVS. The DaM was assumed to be collected from the farms in the form of slurry with 10% TS content. Handling operations and storage of DaM on-farm were outside the system boundaries of the study.

**Table 2:** Compositional data (volatile solids (VS) basis) and biomethane potential (BMP) values for dairy manure used in the present study

Lignin (%VS)	Cellulose (%VS)	Xylose (%VS)	Arabinose (%VS)	Galactose (%VS)	Crude protein (%VS)	Lipid (%VS)	Others (%VS)	VS (%TS)	BMP (mL/gVS)
16.9	32.7	11.9	4.8	2.4	21.2	3.9	31.2	80	211.5

**Fig. 3:**
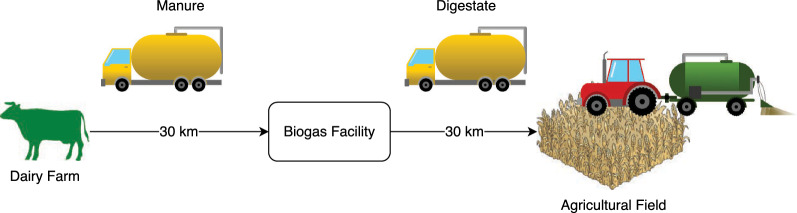
Schematic representation of transport of manure and digestate and field application of digestate

Dairy farms supplying DaM were assumed to be an average distance of 30 km from the biogas plant (Fig. 3). DaM was transported using 40-ton trucks with fuel consumption of 0.74 MJ/tkm, with an empty return trip included [36]. The digestate produced at the end of biogas production was transported to agricultural fields over an average transport distance of 30 km, using the same configuration of 40-ton trucks (Fig. 3). As the digestate was not de-watered, it was assumed that it would be handled similarly to liquid fertiliser. Fuel energy use was determined for transport of DaM and digestate to and from the biogas facility, respectively. The energy needed to spread liquid digestate on agricultural fields was assumed to be 17 MJ per ton of wet digestate at an average spread dose of 30 tons/hectare [37]. Fossil diesel fuel was assumed for all vehicles and machinery involved.

### Process modelling

**Fig. 4:**
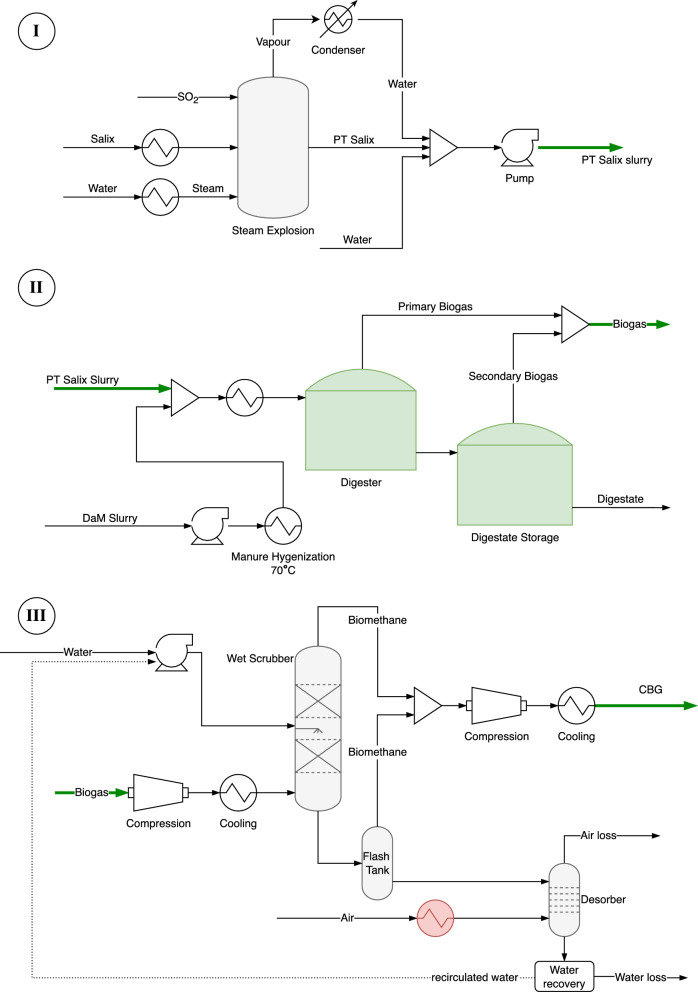
Simplified process flow diagram of stages modelled in Aspen Plus in this study

The energy and mass flows were simulated for stages 2–4 in Fig. 1, comprising steam pre-treatment of *Salix*, co-digestion of *Salix* and hygienized manure to produce biogas, and upgrading of biogas to CBG, using the Aspen Plus process simulation software. Values for the heating, cooling, and electricity energy requirements of these stages were obtained from the Aspen simulation. The facility was designed to process 300 kg of *Salix* dry matter per hour. Dairy manure was added for the co-digestion process, in a 1:1 ratio on a VS basis. The process was modelled in three parts (Fig. 4):

1. Acid-catalysed steam pre-treatment of *Salix* biomass.
2. Anaerobic co-digestion of pre-treated *Salix* and DaM to produce biogas and digestate.
3. Upgrading of biogas to biomethane and compression to CBG.

The process model was adapted from the Aspen model for biodiesel production used by Karlsson et al. [38]. All processes used the NRTL property method in the Aspen simulation.

#### Steam pre-treatment of *Salix*

Steam explosion pre-treatment is one of the most common and efficient pre-treatment methods used commercially for reducing the recalcitrance of lignocellulosic biomass [39, 40]. *Salix* biomass was assumed to be pre-treated by acid-catalysed steam explosion at 185 °C for 4 min, with 2% SO_2_ as catalyst. The pre-treatment conditions were set to be same as those in pre-treatment of the *Salix* samples before BMP assays (SM). The *Salix* pre-treatment process flow analyzed is shown in Part I in Fig. 4. A side-effect of most pre-treatment methods is formation of inhibitory compounds affecting the microorganisms and enzymes responsible for conversion to biofuel [41], and higher pre-treatment severity can lead to increased production of inhibitory compounds [42]. Steam pre-treatment at 180–200 °C for 4–10 min is reported to be favourable for *Salix* [17, 42, 43]. Thus the relatively mild pre-treatment conditions assumed in this study can be expected to minimise the formation of inhibitory compounds. Mild steam pre-treatment primarily affects the hemicellulose content in biomass, and results in the breakdown of polysaccharides (xylan, arabinan, galactan and mannan) to simpler carbohydrates (xylose, arabinose, galactose and mannose). The lignin and cellulose content remains largely unchanged relative to the starting material. Under low-severity pre-treatment conditions, 55–75% of xylan and 60–80% of arabinan are converted [43]. In the steam treatment reactor used in the simulation in this study, conversion of xylan to xylose was assumed to be 60%, and that of arabinan, galactan, and mannan 76%. Composition after pre-treatment of the *Salix* biomasses is shown in Table 3. The steam released after pre-treatment was condensed and added back to the biomass stream. Additional water was assumed to be added to the steam-exploded *Salix* to reach a solids content of 10%, giving a pumpable slurry for the anaerobic digestion process.

**Table 3:** Composition of six varieties of unfertilised (F0) and fertilised (F +) Salix as percentage of total solids (%TS) after steam pre-treatment with 2% SO_2_ at 185 °C for 4 min

Variety	Lignin	Cellulose	Xylan	Xylose	Arabinan	Arabinose	Mannan	Mannose	Galactan	Galactose
Björn F0	24.0	58.7	4.2	6.3	0.1	0.4	0.5	1.5	0.5	1.5
Björn F +	24.2	55.9	4.0	6.0	0.1	0.4	0.4	1.3	0.4	1.3
Gudrun F0	27.3	54.3	4.1	6.1	0.2	0.5	0.4	1.3	0.4	1.3
Gudrun F +	27.9	52.8	3.7	5.5	0.2	0.5	0.4	1.2	0.4	1.4
Jorr F0	27.4	53.5	3.8	5.8	0.2	0.7	0.6	1.9	0.6	2.0
Jorr F +	27.2	51.1	3.5	5.2	0.3	0.8	0.6	1.8	0.5	1.7
Loden F0	28.1	50.9	3.7	5.5	0.2	0.6	0.5	1.5	0.4	1.4
Loden F +	28.8	51.2	3.8	5.6	0.2	0.7	0.5	1.6	0.5	1.4
Tora F0	28.3	51.9	4.2	6.3	0.2	0.6	0.5	1.6	0.5	1.7
Tora F +	26.1	50.1	4.0	6.0	0.2	0.5	0.6	1.8	0.4	1.3
Tordis F0	25.5	57.0	4.0	6.0	0.1	0.4	0.6	1.7	0.5	1.6
Tordis F +	25.7	55.0	4.0	5.9	0.1	0.4	0.5	1.5	0.4	1.3

#### Biogas production

##### Dairy manure hygienisation

DaM was added in a 1:1 VS ratio to the *Salix* biomass and, as the different *Salix* varieties had varying VS content in biomass, the corresponding amount of DaM added to the co-digestion process also changed. There is a known risk of microbiological infection and contamination of the food chain from use of animal manure for production of human and animal feed [44]. Therefore, DaM was assumed to undergo hygienisation at 70 °C for 1 h to reduce the epidemiological risk when digestate from the system was applied to agricultural land. The hygienised DaM stream joined the pre-treated *Salix* stream to produce a combined feedstock slurry, which was fed to the anaerobic digester for biogas production after adjusting to the digester temperature of 37 °C.

##### Anaerobic co-digestion

The anaerobic digester was modelled as a stoichiometric digester in Aspen Plus. A retention time in the digester of 45 days was assumed. Fractional anaerobic conversion of individual components in the digester was determined using biodegradability (BD) ratio as follows: The Buswell equation [45] was used for stoichiometric calculation of anaerobic digestion products from complete conversion of a generic organic material of composition CaHbOcNd, as shown in Eq. 1. Maximum theoretical methane yield (TMY, ml/g VS) was calculated based on the composition of the *Salix* and manure substrates as shown in Eq. 2, using the Buswell equation. While BMP values are a predictor of potential methane production, a direct relationship for prediction of methane production in digesters from BMP values is lacking in the literature [46, 47, 49]. Based on comparative studies [48, 49], real methane yield (RMY) was conservatively estimated to be 80% of the laboratory-scale BMP values. Biodegradability (BD) was defined as the ratio between RMY and TMY (Eq. 3) and determined how much of the substrate is converted into biogas, while the unconverted fraction ended up in the digestate. The TMY, RMY and BD ratios for the different co-digestion mixes of *Salix* varieties and DaM are shown in Table 4. The digester contents were assumed to be agitated with a long-shaft agitator with power consumption of 5.76 kWh/100m^3^/day [50].

**Table 4:** Total methane yield (TMY) and real methane yield (RMY) values for each unfertilised (F0) and fertilised (F +) Salix variety-dairy manure (DaM) co-digestion mix and its biodegradability (BD) ratio

Feedstock	TMY (L/kg feed)	RMY (L/kg feed)	BD (%)
Björn F0 & DaM	63.40	35.38	55.80
Björn F + & DaM	62.82	41.06	65.36
Gudrun F0 & DaM	55.47	37.76	68.0
Gudrun F + & DaM	54.71	37.41	68.38
Jorr F0 & DaM	56.09	35.35	63.03
Jorr F + & DaM	60.25	35.89	59.57
Loden F0 & DaM	54.05	36.77	68.02
Loden F + & DaM	55.17	39.16	70.99
Tora F0 & DaM	57.71	39.68	68.76
Tora F + & DaM	57.35	40.51	70.64
Tordis F0 & DaM	55.71	40.19	72.15
Tordis F + & DaM	57.91	42.32	73.08

1 $${C}_{a}{H}_{b}{O}_{c}{N}_{d}+ \left(a-\frac{b}{4}-\frac{c}{2}+\frac{3d}{4}\right){H}_{2}O \to \left(\frac{a}{2}+\frac{b}{8}-\frac{c}{4}-\frac{3d}{8}\right){CH}_{4}+\left(\frac{a}{2}-\frac{b}{8}+\frac{c}{4}+\frac{3d}{8}\right){CO}_{2}+{dNH}_{3}$$

2 $$TMY=\frac{22.4\times 1000\times \left(\frac{a}{2}+\frac{b}{8}-\frac{c}{4}-\frac{3d}{8}\right)}{12a+b+16c+14d}$$

3 $$BD= \frac{RMY}{TMY}\times 100\%$$

The digestate from the anaerobic digester was assumed to be sent to the digestate storage tank (DST), where further microbial activity was expected to take place, producing a secondary biogas flow. The average temperature of DST was taken to be 20 °C, with HRT of 30 days. Hence, the storage tank was simulated as another stoichiometric digester similar to the anaerobic digester and a further 10% degradation of the remaining organic components was assumed. The biogas from the anaerobic digester and secondary biogas from the DST were sent to the upgrading stage. The digestate stream from the DST was assumed to be pumped to an outlet, after which it was transported via trucks for field application.

#### Upgrading & compression

The upgrading stage was assumed to comprise a water scrubber section to dissolve and remove CO_2_ from the biogas stream, increasing the methane content to more than 95%. This was followed by a compression stage in which biomethane was cooled and compressed at 200 bar and 21 °C to produce CBG. The energy content of the CBG output was calculated using the lower heating value of methane (50 MJ/kg at 25 °C).

#### Potential energy savings – Heat recovery (HRE) scenario

**Fig. 5:**
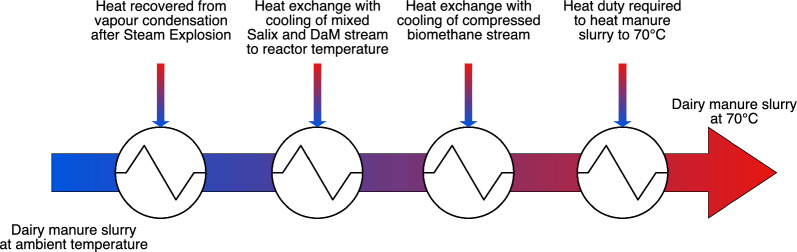
Representation of heat exchanges assumed in the heat recovery (HRE) scenario

The base scenario did not consider any internal heat exchange, with all heating and cooling needs fulfilled with external energy. Stages 2–4 had significant heating and cooling requirements, providing an opportunity to exchange heat between different hot and cold streams to lower the need for external hot and cold utilities. An additional heat recovery (HRE) scenario was designed to reduce the heating demand for hygienisation of DaM slurry. Heat was recovered from three streams within the processes and exchanged with the cold DaM slurry stream as shown in Fig. 5.

### Digester sizing

The volume of the digester (V_d_) was determined from the chosen retention time (T_r_) and daily volumetric input of substrate (S_d_) as:

$${V}_{d}={S}_{d}\times {T}_{r}$$

The *Salix*-DaM slurry fed to anaerobic digester had a solids content of 10%. Due to the high water content of the slurry, a density value of 1 ton/m3 was used to convert the mass flow rate of the slurry to volumetric flow. The volume was calculated based on maximum volumetric flow rate for the different *Salix* and manure combinations. A 45 day retention time gave a digester volume of 7216 m^3^ with an organic loading rate (OLR) of 1.96 kg VS/m3/day (Table 5).

**Table 5:** Size and related parameters of the biogas digester and digestate storage tank (DST)

Maximum daily flow rate of slurry mixture	160.36 tons/day
Max volumetric flow rate of slurry mixture	160.36 m^3^/day
Retention time of digester	45 days
Digester volume	7216 m^3^
Organic loading rate	1.96 kg VS/m^3^/day
Retention time of digestate storage tank	30 days
Digestate storage tank volume	4811 m^3^

### Energy performance calculations

While there is no single standardised method for calculating the energy performance of biogas plants, output–input ratio I is one the most commonly used metrics [51]. Generally, the higher the R value, the better the energy performance of the system. The energy flows included within the input and output categories depend on the system boundaries, and conventions set by the authors of individual studies. The R value of the CBG production system in this study was defined as the ratio of the output energy in the CBG produced (*E*_cbg_) to the secondary energy input in stages 1–5 of the system:

$$R=\frac{{E}_{cbg}}{{E}_{1.f}+{E}_{2-4,h}+{E}_{2-4,c}+{E}_{2-4,el}+{E}_{5,f}}$$

where *E*_*1.f*_ and *E*_*5.f*_ are the fuel (diesel) energy demand of stages 1 and 5, and *E*_2-4,h_, *E*_2-4,c_ and *E*_2-4_,el represent the heating, cooling and electricity inputs in stages 2–4 of the system.

The energy inputs (or demands) were represented in terms of heating, cooling and diesel fuel. The inherent energy contained in the material flows of the feedstocks (*Salix* and DaM) was not included in the input energy, as they were considered to be material inputs to the system undergoing transformation. The *R* value was calculated and is reported for both the base scenario and HRE scenario. The energy used in manufacture and maintenance of infrastructure, vehicles and management was not included in the calculations.

## Results

### Process inputs

The system under study was designed with an input rate of 300 kg/h dry matter of *Salix* biomass. The energy inputs at each stage of the process chain for the base scenario are shown in Table 6 for the different feedstock combinations.

**Table 6:** Hourly energy demand (MJ/h) in each stage and sub-stage of the process chain in the base scenario (without heat recovery) for the different feedstock combinations of Salix varieties and dairy manure (DaM)

	Björn F0 & DaM	Björn F+ & DaM	Gudrun F0 & DaM	Gudrun F+ & DaM	Jorr F0 & DaM	Jorr F+ & DaM	Loden F0 & DaM	Loden F+ & DaM	Tora F0 & DaM	Tora F+ & DaM	Tordis F0 & DaM	Tordis F+ & DaM
**Stage 1**												
*1.1 Salix cultivation and transport*												
Diesel	130.9	214.4	135.2	308.9	141.4	227.7	141.5	331.8	137.3	224.1	131.7	202.8
*1.2 Dairy manure transport*												
Diesel	81.5	81.7	81.4	81.0	81.3	81.7	79.4	80.9	81.0	81.4	81.7	81.7
**Stage 2**												
*2. Pre-treatment*												
Heat	383.8	364.2	371.8	358.8	409.2	407.9	376.4	396.2	390.6	400.2	402.2	382.5
Electricity	1.7	1.7	1.7	1.7	1.7	1.7	1.7	1.7	1.7	1.7	1.7	1.7
**Stage 3**												
*3.1 Manure hygienisation*												
Heat	852.4	854.2	850.9	847.4	850.8	854.0	844.1	847.1	847.6	851.7	854.3	854.9
Electricity	1.11	1.11	1.11	1.10	1.11	1.11	1.10	1.10	1.10	1.11	1.11	1.11
*3.2 Anaerobic Digestion*												
Cooling	246.6	247.0	245.5	243.0	245.7	246.8	241.1	243.1	243.9	245.4	247.8	247.5
Electricity	62.2	62.2	62.2	62.2	62.2	62.2	62.2	62.2	62.2	62.2	62.2	62.2
**Stage 4**												
*4.1 Upgrading*												
Heat	20.3	20.3	20.3	20.3	20.3	20.3	20.3	20.3	20.3	20.3	20.3	20.3
Cooling	95.4	104.5	107.4	105.1	100.6	93.4	103.0	107.5	106.4	106.4	115.5	113.8
Electricity	124.8	133.4	136.1	133.9	129.7	122.8	131.9	136.1	135.1	135.1	143.7	142.1
*4.2 Compression*												
Cooling	139.1	150.1	153.4	150.6	145.2	136.3	147.9	153.4	152.1	152.1	163.4	161.2
Electricity	138.7	149.6	153.0	150.1	144.8	135.9	147.5	152.9	151.7	151.6	163.0	160.8
**Stage 5**												
*5.1 Digestate transport*												
Diesel	149.7	149.3	148.8	148.6	149.2	150.0	147.1	148.4	148.6	149.0	148.6	148.8
*5.2 Digestate spreading*												
Diesel	114.6	114.3	114.0	113.8	114.3	114.8	112.7	113.7	113.8	114.1	113.8	114.0

Energy demand as diesel in cultivation and transport was higher for all fertilised varieties compared with their unfertilised counterparts. This was due to the additional energy usage in production and application of fertilisers to fields. However, as fertilisation usually results in a greater amount of shoot biomass, fertilised *Salix* requires less land per unit mass of biomass produced. Reported average land area required to produce a ton of *Salix* biomass varies from 0.06 ha for the highly productive variety Tordis to 0.21 ha for the low-producing Jorr and Loden [28]. There was slight variation in amount of DaM added to the different *Salix* feedstock mixtures in this study as VS % differed between the varieties and the two feedstocks were combined in a 1:1 VS ratio. This led to minor variations in the energy demand for transportation of DaM.

The biogas facility encompassed stages 2–4, i.e., steam pre-treatment of *Salix*, manure hygienisation and anaerobic digestion, and upgrading of biogas to compressed biomethane. The energy demands of these stages were obtained from the process model created in Aspen Plus. The average energy flows of modelled unit processes are shown in Additional file 1: Table S5. The inputs in the pre-treatment phase varied slightly between the different *Salix* varieties, as the net mass of biomass treated was the same, but with some variations in composition. A large amount of heat was required in the steam explosion process as a result of production of superheated steam. The hygienisation process was the most energy-intensive step in the entire process chain, due to the high energy demand for heating DaM to 70 °C. The hygienised manure was mixed with the pre-treated *Salix* slurry and the combined feedstock stream needed cooling to the digester operating temperature of 37°C before anaerobic digestion.

Deviations in the composition of feedstocks and in BD between the manure and *Salix* mixtures resulted in variations in the amount of biogas generated and its composition. Higher BD led to greater conversion of organic matter to biogas, leading to greater flow rates of biogas. Higher amount of biogas produced meant that more electricity and cooling were required in the upgrading and compression steps. The water used in wet scrubbing of biogas to remove CO_2_ was recirculated with a loss of 3%, reducing the need for addition of fresh water. The heat demand in the upgrading stage was for heating air used to remove dissolved gases from the water, which was then released from the system. Since compression of gases generates heat, the compressed gases needed to be cooled between stages, leading to high cooling demand in Stage 4.

Transportation of digestate to agricultural farms and spreading of digestate were performed using machines with diesel fuel as their energy source. Diesel energy demand for these activities was similar between the different varieties.

### Biogas output

There were large variations in simulated biogas yield between the different *Salix* feedstock combinations studied because of the variation in composition and BMP, as reflected in the BD ratios. Primary and secondary biogas flows produced in the biogas digester and DST were upgraded and compressed to CBG. Feedstock mixtures with the varieties Gudrun and Tordis were the most productive CBG producers, while var. Jorr was the least productive (Table 7). In terms of CBG produced per unit of VS in the system, fertilised Jorr and unfertilised Björn showed lowest conversion of VS to the final product (Table 7).

**Table 7:** Biogas and compressed biomethane gas (CBG) yield on an hourly basis from the anaerobic digester and digestate storage tank (DST) (F0 & F + indicate unfertilised and fertilised Salix, respectively, DaM is dairy manure)

Feedstock	Biogas flow (kg/h)		Compressed biomethane gas flow		
	DIGESTER	DST	Kg/h	Nm^3^/h	kg/kg VS
Björn F0 & DaM	228	15	85	118	0.14
Björn F + & DaM	256	12	92	128	0.16
Gudrun F0 & DaM	264	11	94	131	0.16
Gudrun F + & DaM	259	11	92	128	0.16
Jorr F0 & DaM	278	14	89	124	0.15
Jorr F + & DaM	224	13	83	116	0.14
Loden F0 & DaM	254	11	90	126	0.16
Loden F + & DaM	266	10	94	131	0.16
Tora F0 & DaM	262	11	93	130	0.16
Tora F + & DaM	262	10	93	130	0.16
Tordis F0 & DaM	288	10	100	139	0.17
Tordis F + & DaM	283	10	99	137	0.17

### Annual energy balance

The annual energy inputs and outputs (energy contained in the final CBG) with an annual operating time of 8000 h for the two scenarios are shown in Table 8. The energy performance was calculated based on energy output–input ratio (R).

**Table 8:** Energy balance and energy ratio (R) of unfertilised (F0) and fertilised (F +) Salix varieties and dairy manure (DaM) co-digestion feedstocks in the biogas system under base and heat recovery (HRE) scenario

Feedstock	Björn F0 & DaM	Björn F + & DaM	Gudrun F0 & DaM	Gudrun F + & DaM	Jorr F0 & DaM	Jorr F + & DaM	Loden F0 & DaM	Loden F + & DaM	Tora F0 & DaM	Tora F + & DaM	Tordis F0 & DaM	Tordis F + & DaM	Unit
Net energy output^*^	9.43	10.19	10.42	10.22	9.85	9.24	10.04	10.41	10.33	10.32	11.10	10.95	GWh
Base scenario													
Net diesel demand	1.06	1.24	1.07	1.45	1.08	1.28	1.07	1.50	1.07	1.26	1.06	1.22	GWh
Net heating demand	2.79	2.75	2.76	2.73	2.85	2.85	2.76	2.81	2.80	2.83	2.84	2.79	GWh
Net cooling demand	1.07	1.11	1.13	1.11	1.09	1.06	1.09	1.12	1.12	1.12	1.17	1.16	GWh
Net electricity demand	0.73	0.77	0.79	0.78	0.75	0.72	0.77	0.79	0.78	0.78	0.83	0.82	GWh
Output–input ratio	1.67	1.73	1.81	1.69	1.71	1.57	1.77	1.68	1.79	1.72	1.88	1.83	
HRE scenario													
Net diesel demand	1.06	1.24	1.07	1.45	1.08	1.28	1.07	1.50	1.07	1.26	1.06	1.22	GWh
Net heating demand	1.62	1.56	1.56	1.54	1.66	1.68	1.56	1.61	1.60	1.63	1.61	1.57	GWh
Net cooling demand	0.24	0.26	0.27	0.26	0.25	0.23	0.26	0.27	0.26	0.26	0.29	0.28	GWh
Net electricity demand	0.73	0.77	0.79	0.78	0.75	0.72	0.77	0.79	0.78	0.78	0.83	0.82	GWh
Output–input ratio	2.59	2.66	2.83	2.54	2.63	2.36	2.75	2.50	2.78	2.62	2.94	2.82	
Improvement in HRE scenario over base scenario	55.1%	53.6%	56.0%	50.6%	54.2%	51.0%	55.5%	49.2%	55.1%	52.1%	55.9%	54.0%	

All *Salix* and DaM-based co-digestion systems had a R value greater than 1, which means that more energy was obtained in the final product (biomethane) than was demanded in the complete system. Unfertilised Tordis-based systems had the highest R value (1.88), while fertilised Jorr had the lowest (1.57). Most of the energy demand was in the form of heating, which can be attributed to the large energy requirement for hygienisation of DaM and steam explosion of *Salix* biomass. As the manure had a moisture content of 90%, a significant amount of energy was required to heat it to 70°C to reduce the risk of pathogen contamination from field application of digestate. The cooling demand was also high, indicating potential for heat exchange between the heating and cooling functions to reduce the overall demand of the facility.

Under the HRE scenario, there was a significant reduction in energy demand for heating and cooling owing to heat exchange between selected hot streams and the cold DaM feed stream (Table 8). Heat recovery did not affect the energy output in terms of CBG, which remained the same as in the base scenario. This led to improved energy performance in the HRE scenario, resulting in higher *R* values for all cases (Table 8). Energy performance improved by 46–61% in the heat recovery scenario compared with the base scenario. Biomethane production from co-digestion of unfertilised Tordis with DaM had the highest R value in the HRE scenario (2.94), while the fertilised Jorr with DaM system had the lowest (2.36).

### Energy demand by process

**Fig. 6:**
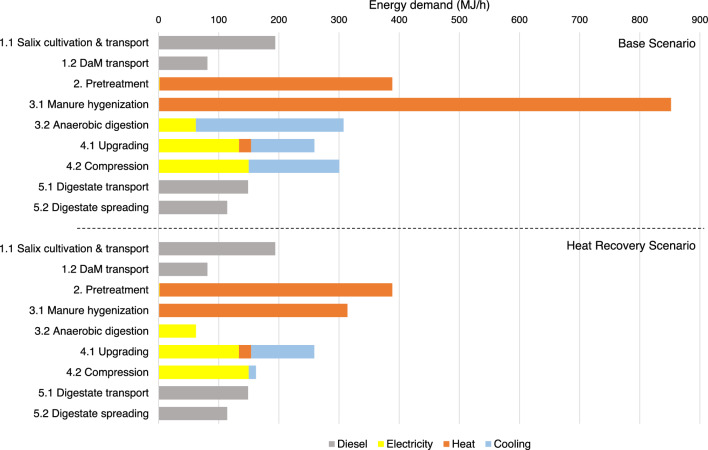
Average energy demand (diesel, electricity, heating and cooling) of the different processes in the compressed biomethane gas (CBG) production chain for the different Salix varieties and dairy manure (DaM) co-digestion feedstocks

The average energy requirement by type as diesel, electricity, heat and cooling across the different processes in the whole production chain for the different feedstock cases are presented in Fig. 6. In the base scenario, manure hygienisation and steam pre-treatment had the largest energy demand in the form of heating. The HRE scenario greatly reduced the heat demand for manure hygienisation. Diesel energy demand was sensitive to transportation distance, especially in the case of digestate disposal, as transportation of large volumes of wet digestate over greater distances greatly increased the diesel energy demand. Thus longer transport distances will require alternate strategies for the digestate to maintain desirable energy performance.

## Discussion

This study analyzed the effects of *Salix* variety on energy and mass flows co-digested with DaM to produce biomethane. The results from the literature, laboratory experiments and process modelling were useful in identifying factors and parameters affecting energy output and performance of the anaerobic digestion process. Overall, the results showed good potential for biomethane production and can serve as a guideline for future assessments to determine biomethane output in relation to amount of *Salix* processed. Site-specific data that include spatial and temporal aspects are needed to refine the results further to provide exact figures for real biogas applications.

The results in the present case showed that the energy output was higher than the energy demand of the *Salix*-to-biomethane systems, but with differences between varieties, highlighting the importance of including varietal effects in such analyses. The wide system boundary chosen in the study (Fig. 1) also provided a more holistic picture of the performance of the system, as all steps from cultivation of *Salix* to digestate disposal were included.

There were large variations in energy demand of the *Salix* production chain between the different varieties, due to fertilisation and differences in yield. Fertilisation increased the energy demand per unit mass of biomass produced, but also gave higher biomass yield in most cases. The productivity of *Salix* crop varieties is an important parameter, as there is reported to be a 3.5-fold difference in land requirement between the lowest- and highest-producing varieties [28]. Arable land is a scarce resource in the majority of countries worldwide, so it is important to strike a balance between the amount of land needed for production and the energy input per unit of biomass. The productivity level of unfertilised crops is also questionable in the long run, as it is very likely that the soil nutrients will deplete over time. Thus, fertilisation is beneficial to ensure a steady and secure supply of *Salix* biomass.

The biomethane facility studied comprised steam pre-treatment, hygienisation of manure and anaerobic digestion, and biogas upgrading (stages 2, 3 and 4 in Fig. 1). The hygienisation process had the highest energy demand, for heating liquid dairy manure to deactivate pathogens. This increased the energy input of the system, but rendered the digestate safe as a fertiliser. Although optimisation of energy performance is important, it is not always the main objective of biogas plants. Use of digestate on fields reduces the need for mineral fertilisers and can contribute to increased soil carbon sequestration. This is favourable from the perspective of climate change mitigation and waste management. Climate impact studies on the system scale (e.g., LCA) are needed to calculate the climate benefit of such processes. Based on the N-P-K content of *Salix* biomass and DaM reported in the literature (Additional file 1: Table S6), annual application of 30 tons/hectare of digestate can add about 60 kg, 12.5 kg and 59 kg of N-P-K per year (Additional file 1: Table S7).

Co-digestion of the *Salix* varieties Gudrun and Tordis with DaM gave the highest biomethane output in this study. In both fertilised and unfertilised form, these two varieties produced more than 100 kg/h of biomethane from co-digestion of 300 kg/h of *Salix* feedstock with DaM in a 1:1 VS ratio. The biomethane output was modelled in reactors in Aspen Plus, using stoichiometric reactions and BD ratio calculated from laboratory-scale BMP studies. Different approaches in modelling biogas reactors can lead to varying results and there is uncertainty regarding how biogas production in industrial-scale plants compares with laboratory-scale experiments. Anaerobic digestion is a simple process but has complex dynamics, as it involves intricate microbiological interactions, so it is difficult to upscale laboratory-scale BMP values to methane production in large-scale plants. In this study, RMY was conservatively assumed to be 80% of the BMP value. Depending on anaerobic digester conditions and management practices, RMY can be higher. Liquid digestate recirculation could be an interesting strategy to increase biomass degradability and reach higher methane yields as some studies as reported [52, 53]. For instance, liquid digestate could be utilized instead of water to increase water content of the pretreated *Salix* biomass to make it pumpable. Experimentation is required to determine optimal recirculation ratios for the feedstocks studied and to avoid negative effects such as inhibitor accumulation or accumulation of solids. Pilot-scale studies are needed to identify the reaction dynamics and interactions, which will allow more accurate modelling and extrapolation of such processes to industrial scales.

Due to the lack of a standardised method for measuring energy performance, it is challenging to make direct comparisons of different systems. Output–input ratio is one of the most common indicators used in energy performance calculations for biogas production [51], but differences in system boundaries between studies determine what are included as input and output energies in respective systems. To conduct an accurate energy balance analysis, direct and indirect energy requirements should be established for all stages of the crop-based energy production cycle. The *R* values in this study ranged from 1.57 to 1.88 for the base scenario without heat recovery, and from 2.36 to 2.94 in the heat recovery scenario. These values are at the lower end of the range of R values reported in the literature, e.g., for *Salix* biogas production in Denmark values of 7.3 without pre-treatment and 12.3 with pre-treatment have been reported [54]. Those higher R values can be due to omission of biomethane upgrading and manure hygienisation processes in their system. The *R* values in that study were higher for *Salix* than for maize and miscanthus, although total energy output was higher from maize without pre-treatment. The perennial energy crops (*Salix* and miscanthus) had significantly lower energy inputs for cultivation and harvest than maize, and pre-treatment improved biogas yield [54]. A similar analysis of biomethane production from untreated hemp (*Cannabis sativa* L.) in Sweden reported a R value of 2.6 [55], which is comparable to the R values of *Salix* in the HRE scenario in our study. Another study analysing biomethane production from maize, fodder beet, lupin and perennial ryegrass, with heat and electricity demand fulfilled from the biogas produced, reported *R* values of 2.0 to 2.9 for crops in the system [54]. However, the results of such systems analyses are dependent on site-specific conditions and modelling choices such as system configuration, secondary feedstock selection, and pretreatment conditions. Hence, it is important to consider these factors when interpreting the results.

The heating demand for the pretreatment and manure hygienization processes is one of the main energy consumers in the system. Reduction of the pretreatment energy consumption for pre-treatment, while maximising the release of sugars, is critical for improving the energy performance of biomass to biofuel systems [56]. Achieving such an improvement could make lignocellulosic materials such as *Salix* an efficient and attractive feedstock for sustainable production of biofuels and biogas [57]. The lower energy demand in the HRE scenario improved the energy performance of the system in this study. Process design to maximise heat recovery while balancing the economic costs of a more complex set-up is necessary to ensure the success of industrial-scale production.

The heating value of the raw materials was not considered in this study, as the energy performance of different energy carriers other than biomethane or other conversion pathways (e.g., combustion or gasification) for the feedstocks were not compared. The focus was on biomethane production and the system performance of different feedstock combinations. Differences in energy conversion efficiency must be included when comparing different conversion pathways.

Upgrading and compression of biogas to biomethane had a high demand for electricity and cooling, which negatively affected the overall energy balance, as these steps did not increase the net energy output of the system. The increased energy demand for upgrading biogas can be justified, as it improves fuel quality and enables direct use of biogas as a vehicle fuel or injection into gas grids as biomethane. If biogas is to replace natural gas as fuel, upgrading is necessary to remove the non-combustible CO_2_ fraction from biogas. Reducing the energy demand for upgrading would greatly benefit the energy performance of the system, but might not be as relevant from an economic standpoint if cheap electricity and cooling are available on-site. Various upgrading technologies (in addition to water scrubbing) are undergoing constant improvement in their energy and environmental performance, but their actual performance will depend on site-specific and economic conditions, which must be taken into account when selecting the best technique [58].

In addition to replacement of fossil natural gas by biomethane, potential for soil carbon sequestration by *Salix* cultivation [25] and digestate application [59] make co-digestion of *Salix* an interesting strategy to mitigate climate change. In future work, we will extend the mass and energy analysis to a LCA to evaluate and compare the climate performance of biomethane production from *Salix* varieties.

## Conclusions

A CBG production system based on a 1:1 VS mix of pre-treated *Salix* and DaM was analyzed to evaluate the energy performance of different *Salix* varieties. Biomethane production varied between different combinations of *Salix* and DaM, based on BMP values and composition. The energy demand of the biomethane production chain in terms of heating, cooling, and electricity demand was assessed in scenarios without and with heat recovery. Output–input ratio varied from 1.57 to 1.88 in the scenario without heat recovery, while including heat recovery to meet some of the heating and cooling requirements increased the *R* value to 2.36–2.94. A system based on unfertilised var. Tordis performed best, fertilised Jorr was the worst in both scenarios. The hygienization of DaM was the greatest contributor to the heating demand, followed by upgrading and compression of biogas to biomethane. The heat recovery scenario greatly reduced the energy demand; however, upgrading still represented a high energy demand owing to the higher electricity demand. A reduction in the energy required for upgrading can significantly improve energy performance. The energy performance showed that, *Salix* could be a potential feedstock for biogas production, although its *R* value was at the lower end of the reported range for biogas from energy crops. However, direct comparison between studies is difficult due to differences in system boundaries and conditions. Further work will focus on determining the climate impacts of these *Salix*-based biomethane systems, to assess their potential to mitigate climate change.

### Supplementary Information

**Additional file 1:** **Table S1.** Composition data for the six Salix varieties under unfertilised (F0) and fertilised (F+) conditions. Values are based on total solids content. Values are means of three biological replicates. **Table S2.** Average yield of Salix varieties in tons (t) of dry matter (DM) per hectare (ha) per 3-year harvest cycle and annual average [5]. **Table S3. **Material inputs per hectare in Salix cultivation. Cuttings and pesticide were used during establishment of a new rotation every 25 years. Fertilisers were applied annually from the second year of establishment. The values were obtained from a field study by Weih and Nordh [6] at Uppsala, Sweden. **Table S4. **Energy input in terms of diesel fuel for processes involved in Salix cultivation per ton dry matter (t DM) of harvested biomass [5]. **Table S5.** Energy in terms of electricity, heating and cooling for the unit processes modelled using Aspen Plus. **Table S6.** Nitrogen (N), Phosphorus (P) and Potassium (K) content (% in dry matter) for Salix shoot biomass (dry) for fertilised and unfertilised varieties, and manure. **Table S7.** Amounts of N P K added and area needed for digestate spreading when digestate is spread at a rate of 30 tons/hectare annually.

## Data Availability

The data presented in this study are available the supplementary material and within the article. If required, further relevant details are available from the corresponding author on request.

## Abbreviations

BD: Biodegradability
BMP: Biomethane potential
CBG: Compressed biomethane gas
DaM: Dairy manure
DST: Digestate storage tank
EU: European Union
F+: Fertilised
F0: Unfertilised
GDP: Gross domestic product
GHG: Greenhouse gas
HRT: Hydraulic retention time
IEA: International Energy Agency
IPCC: Intergovernmental Panel on Climate Change
LCA: Life cycle assessment
NRTL: Non-random two liquid
OLR: Organic loading rate
PPP: Purchasing power parity
RMY: Real methane yield
SM: Supplementary material
TMY: Theoretical methane yield
TS: Total solids
VS: Volatile solids
