# Supplementary material for: Energy performance of compressed biomethane gas production from co-digestion of Salix and dairy manure: factoring differences between Salix varieties
Source: Biotechnol Biofuels Bioprod. 2023 Nov 3;16:165. doi: 10.1186/s13068-023-02412-1 (PMC10625243; doi:10.1186/s13068-023-02412-1)
Supplement: Supplementary file 1 — Additional file 1: Table S1. Composition data for the six Salix varieties under unfertilised (F0) and fertilised (F+) conditions. Values are based on total solids content. Values are means of three biological replicates. Table S2. Average yield of Salix varieties in tons (t) of dry matter (DM) per hectare (ha) per 3-year harvest cycle and annual average [5]. Table S3. Material inputs per hectare in Salix cultivation. Cuttings and pesticide were used during establishment of a new rotation every 25 years. Fertilisers were applied annually from the second year of establishment. The values were obtained from a field study by Weih and Nordh [6] at Uppsala, Sweden. Table S4. Energy input in terms of diesel fuel for processes involved in Salix cultivation per ton dry matter (t DM) of harvested biomass [5]. Table S5. Energy in terms of electricity, heating and cooling for the unit processes modelled using Aspen Plus. Table S6. Nitrogen (N), Phosphorus (P) and Potassium (K) content (% in dry matter) for Salix shoot biomass (dry) for fertilised and unfertilised varieties, and manure. Table S7. Amounts of N P K added and area needed for digestate spreading when digestate is spread at a rate of 30 tons/hectare annually. [file 13068_2023_2412_MOESM1_ESM.docx]

## Additional file 1

## Salix Compositional Analysis Data

The milled and sieved biomass samples were Soxhlet extracted according to the NREL procedure TP-510-42619. Samples underwent extraction with boiling water for 6 hours and ethanol (95%) for an additional 6 hours. All extracted samples were then dried, and the monomeric carbohydrates contents of the samples were determined by quantitative saccharification upon acid hydrolysis and subsequent HPLC analysis, based on the NREL procedure 510-42618. The sugar compositions were determined by HPLC (Chromaster) equipped with an evaporative light scattering detector (ELSD-go), and a Metacarb 87P column operated at 80°C. Ohlsson, 2021 [1], presents the details of the compositional analysis. Mohamed Jebrane at Department of Forest Biomaterials and Technology, SLU carried out the compositional analysis tests.

## Biomethanation Potential Assays

Biomass was chipped using a compost chipper (MTD90 Chipper Shredder). The chipped material was steam pretreated using relatively mild conditions (185 °C for 4 minutes, 2% SO_2_ as a catalyst), equalling a severity factor (log_10_ R_0_) of 3.1. The steam pretreatment was performed in a 10-L reactor (Process & Industriteknik AB, Kristianstad, Sweden) described previously by Palmqvist et al. (1996) [2]. The material was stored in –20°C before and after steam pretreatment, and thawed at 4°C prior to BMP assays.

The BMP assay was performed using steam pretreated Salix biomass. In 1120-ml serum bottles, 1.2 g volatile solids (VS) were mixed with inoculum from a wastewater treatment plant in Uppsala, Sweden. Inoculum to substrate ratio was 3:1 on a VS basis, and tap water was added to reach a final liquid volume of 400 ml. Total solids (TS) and VS were measured by drying at 105°C followed by incineration at 550°C. Inoculum TS was 3.7% and VS was 2.4%, based on wet weight. Bottles were sealed with butyl rubber seals and aluminium caps and incubated at 37°C on a rotary shaker set to 100 rpm. Gas production was evaluated manometrically, and methane contents analysed using gas chromatography as previously described by [3], [4] . Triplicate cellulose (1.2 g VS per bottle; medium fibers; Sigma-Aldrich) and inoculum controls were included in the assay. Due to very low BMP values, indicating an issue with the assay, the Jorr F+ samples were re-evaluated on an AMPTS system using the same parameters as above.

Table S1 Composition data for the six Salix varieties under unfertilised (F0) and fertilised (F+) conditions. Values are based on total solids content. Values are means of three biological replicates.

| Variety & treatment | Lignin (%) | Cellobiose (%) | Glucose (%) | Xylose (%) | Galactose (%) | Arabinose (%) | Mannose (%) |
| --- | --- | --- | --- | --- | --- | --- | --- |
| Björn F0 | 24.0 | 0.1 | 58.6 | 10.4 | 2.0 | 0.5 | 2.0 |
| Björn F+ | 24.2 | 0.1 | 55.8 | 9.9 | 1.7 | 0.5 | 1.7 |
| Gudrun F0 | 27.3 | 0.0 | 54.3 | 10.2 | 1.8 | 0.7 | 1.8 |
| Gudrun F+ | 27.9 | 0.1 | 52.7 | 9.2 | 1.8 | 0.7 | 1.6 |
| Jorr F0 | 27.4 | 0.7 | 52.8 | 9.6 | 2.6 | 0.9 | 2.4 |
| Jorr F+ | 27.2 | 0.3 | 50.7 | 8.7 | 2.2 | 1.0 | 2.4 |
| Loden F0 | 28.1 | 0.1 | 50.8 | 9.2 | 1.8 | 0.7 | 1.9 |
| Loden F+ | 28.8 | 0.1 | 51.1 | 9.4 | 1.9 | 0.9 | 2.2 |
| Tora F0 | 28.3 | 0.1 | 51.8 | 10.4 | 2.3 | 0.8 | 2.1 |
| Tora F+ | 26.1 | 0.0 | 50.1 | 10.0 | 1.8 | 0.7 | 2.3 |
| Tordis F0 | 25.5 | 0.1 | 57.0 | 10.1 | 2.1 | 0.6 | 2.3 |
| Tordis F+ | 25.7 | 0.0 | 55.0 | 9.9 | 1.7 | 0.5 | 2.0 |

## Input Data for Salix Cultivation and harvest

Table S2 Average yield of Salix varieties in tons (t) of dry matter (DM) per hectare (ha) per 3-year harvest cycle and annual average [5]

| Variety and treatment | Harvest (t DM/ha) | |
| --- | --- | --- |
|  | 3-year rotation | Annual average |
| Björn_F0 | 31.9 | 10.6 |
| Björn_F+ | 42.7 | 14.2 |
| Gudrun_F0 | 20.8 | 6.9 |
| Gudrun_F+ | 20.6 | 6.9 |
| Jorr_F0 | 14.4 | 4.8 |
| Jorr_F+ | 36.9 | 12.3 |
| Loden_F0 | 14.4 | 4.8 |
| Loden_F+ | 18.3 | 6.1 |
| Tora_F0 | 18.2 | 6.1 |
| Tora_F+ | 38.3 | 12.8 |
| Tordis_F0 | 28.5 | 9.5 |
| Tordis_F+ | 48.5 | 16.2 |

Table S3 Material inputs per hectare in Salix cultivation. Cuttings and pesticide were used during establishment of a new rotation every 25 years. Fertilisers were applied annually from the second year of establishment. The values were obtained from a field study by Weih and Nordh [6] at Uppsala, Sweden.

| Input | Value | Unit per hectare | |  |
| --- | --- | --- | --- | --- |
| Cuttings^1^ | 18000 | Cuttings/rotation | |  |
| Pesticide |  |  | |  |
| Roundup | 5 | l/rotation | |  |
| Cougar | 1 | l/rotation | |  |
| Mineral Fertiliser | | |  | |
| N | 100 | kg/year | |  |
| P | 14 | kg/year | |  |
| K | 47 | kg/year | |  |

^1^Salix was planted at a density of 18000 cuttings per hectare

Table S4 Energy input in terms of diesel fuel for processes involved in Salix cultivation per ton dry matter (t DM) of harvested biomass [5]

| Variety & treatment | Pesticides | Field preparation | Seedling production & planting | Fertiliser production and application | Harvest and chipping | Forwarding | Stump removal | Transport to biogas facility |
| --- | --- | --- | --- | --- | --- | --- | --- | --- |
|  | MJ/ t DM | MJ/ t DM | MJ/ t DM | MJ/ t DM | MJ/ t DM | MJ/ t DM | MJ/ t DM | MJ/ t DM |
| Björn F0 | 7.0 | 12.2 | 7.5 | - | 120.2 | 58.7 | 2.9 | 227.7 |
| Björn F+ | 5.1 | 8.9 | 5.5 | 286.3 | 120.2 | 58.7 | 2.1 | 227.7 |
| Gudrun F0 | 10.5 | 18.2 | 11.1 | - | 120.2 | 58.7 | 4.4 | 227.7 |
| Gudrun F+ | 10.4 | 18.1 | 11.1 | 579.2 | 120.2 | 58.7 | 4.3 | 227.7 |
| Jorr F0 | 15.3 | 26.7 | 16.3 | - | 120.2 | 58.7 | 6.4 | 227.7 |
| Jorr F+ | 5.9 | 10.2 | 6.3 | 327.5 | 120.2 | 58.7 | 2.5 | 227.7 |
| Loden F0 | 15.4 | 26.8 | 16.4 | - | 120.2 | 58.7 | 6.4 | 227.7 |
| Loden F+ | 11.7 | 20.3 | 12.4 | 650.0 | 120.2 | 58.7 | 4.9 | 227.7 |
| Tora F0 | 12.1 | 21.0 | 12.9 | - | 120.2 | 58.7 | 5.0 | 227.7 |
| Tora F+ | 5.7 | 9.9 | 6.0 | 316.5 | 120.2 | 58.7 | 2.4 | 227.7 |
| Tordis F0 | 7.7 | 13.4 | 8.2 | - | 120.2 | 58.7 | 3.2 | 227.7 |
| Tordis F+ | 4.5 | 7.8 | 4.8 | 250.5 | 120.2 | 58.7 | 1.9 | 227.7 |

## Energy of unit processes determined from process model

The electricity, heating and cooling duties in Table 7 were obtained from the process models created using Aspen Plus V11.

Table S5 Energy in terms of electricity, heating and cooling for the unit processes modelled using Aspen Plus

| **Unit process** | **Electricity req.** | **Heat req.** | **Cooling req.** |
| --- | --- | --- | --- |
| ***Pretreatment*** |  |  |  |
| Salix heating |  | 7 kW/h |  |
| High pressure steam generation |  | 39 kW/h |  |
| Steam Explosion |  | 54 – 68 kW/h |  |
| SO2 pump | 0.18 kW/h |  |  |
| Water pump | 0.28 kW/h |  |  |
| Recoverable heat from steam post  steam explosion |  |  | 42.6 kW/h |
| ***Anaerobic Digestion*** |  |  |  |
| Manure Hygenization |  | 234 – 237 kW/h  (base scenario)  84 – 91 kW/h  (heat recovery scenario) |  |
| Manure pump | 0.31 kW/h |  |  |
| Excess heat in digestate (Cooling) |  |  | 67 – 69 kW/h  (base scenario) |
| Digestate pump | 2 kW/h |  |  |
| ***Upgrading*** |  |  |  |
| Compressor block 1 | 17 – 21 kW/h |  | 14 – 17 kW/h |
| Compressor block 2 | 7 – 9 kW/h |  | 12 – 15 kW/h |
| Air heating |  | 5.63 kW/h |  |
| Water pump | 10 kW/h |  |  |
| ***Compression*** |  |  |  |
| Compression block 3 | 38 – 45 kW/h |  | 38 – 46 kW/h  (base scenario)  29 – 36 kW/h (heat recovery scenario) |

## Nutrient (N-P-K) content in feedstock and digestate

Table S6 Nitrogen (N), Phosphorus (P) and Potassium (K) content (% in dry matter) for Salix shoot biomass (dry) for fertilised and unfertilised varieties, and manure

|  | Treatment | N (%) | P (%) | K (%) |
| --- | --- | --- | --- | --- |
| Björn | F0 | 0.19 | 0.08 | 0.27 |
| Björn | F+ | 0.19 | 0.08 | 0.27 |
| Gudrun | F0 | 0.20 | 0.08 | 0.27 |
| Gudrun | F+ | 0.25 | 0.08 | 0.27 |
| Jorr | F0 | 0.22 | 0.08 | 0.27 |
| Jorr | F+ | 0.18 | 0.08 | 0.27 |
| Loden | F0 | 0.23 | 0.08 | 0.27 |
| Loden | F+ | 0.29 | 0.08 | 0.27 |
| Tora | F0 | 0.21 | 0.08 | 0.27 |
| Tora | F+ | 0.19 | 0.08 | 0.27 |
| Tordis | F0 | 0.22 | 0.08 | 0.27 |
| Tordis | F+ | 0.23 | 0.08 | 0.27 |
| DaM |  | 3.5 | 0.7 | 3.4 |

Nitrogen content in Salix shoot biomass is based on data from field study by Weih and Nordh [6], and the phosphorus and potassium content is estimated by data from Phyllis database [7]. NPK content of DaM is based on mean of literature values [8]–[13]. The N P K amounts in the mixed feed of Salix and DaM is assumed to end up in the digestate in calculation of the nutrient content of the digestate. Considering an annual digestate application rate of 30 tons/hectare, the potential NPK added to per hectare of land and the annual area needed to spread this digestate are calculated and presented in Table 6.

Table S7 Amounts of N P K added and area needed for digestate spreading when digestate is spread at a rate of 30 tons/hectare annually

| Digestate | Treatment | N (kg/ha) | P (kg/ha) | K (kg/ha) | Area needed (ha) |
| --- | --- | --- | --- | --- | --- |
| Björn + DaM | F0 | 59.7 | 12.5 | 59.1 | 1968.8 |
| Björn + DaM | F+ | 60.0 | 12.5 | 59.4 | 1963.8 |
| Gudrun + DaM | F0 | 60.1 | 12.5 | 59.4 | 1957.3 |
| Gudrun + DaM | F+ | 60.6 | 12.5 | 59.2 | 1954.7 |
| Jorr + DaM | F0 | 60.2 | 12.5 | 59.2 | 1962.7 |
| Jorr + DaM | F+ | 59.6 | 12.5 | 59.1 | 1972.5 |
| Loden + DaM | F0 | 60.7 | 12.6 | 59.6 | 1935.4 |
| Loden + DaM | F+ | 61.2 | 12.5 | 59.3 | 1952.5 |
| Tora + DaM | F0 | 60.1 | 12.5 | 59.3 | 1954.0 |
| Tora + DaM | F+ | 59.9 | 12.5 | 59.4 | 1959.2 |
| Tordis + DaM | F0 | 60.7 | 12.6 | 59.7 | 1955.1 |
| Tordis + DaM | F+ | 60.8 | 12.6 | 59.7 | 1957.3 |

# References

[1] J. Ohlsson, “Salix as a biorefinery feedstock : an inquiry into factors affecting conversion performance,” Doctoral Thesis, Swedish University of Agricultural Sciences (SLU), Uppsala, 2021. Accessed: Jan. 07, 2023. [Online]. Available: https://res.slu.se/id/publ/111794

[2] E. Palmqvist *et al.*, “Design and operation of a bench-scale process development unit for the production of ethanol from lignocellulosics,” *Bioresource Technology*, vol. 58, no. 2, pp. 171–179, Nov. 1996, doi: 10.1016/S0960-8524(96)00096-X.

[3] M. Westerholm, S. Roos, and A. Schnürer, “Syntrophaceticus schinkii gen. nov., sp. nov., an anaerobic, syntrophic acetate-oxidizing bacterium isolated from a mesophilic anaerobic filter,” *FEMS Microbiology Letters*, vol. 309, no. 1, pp. 100–104, Aug. 2010, doi: 10.1111/j.1574-6968.2010.02023.x.

[4] J. A. Ohlsson, A. E. Harman-Ware, M. Sandgren, and A. Schnürer, “Biomass Recalcitrance in Willow Under Two Biological Conversion Paradigms: Enzymatic Hydrolysis and Anaerobic Digestion,” *Bioenerg. Res.*, vol. 13, no. 1, pp. 260–270, Mar. 2020, doi: 10.1007/s12155-019-10079-6.

[5] S. Kalita, H. K. Potter, M. Weih, C. Baum, Å. Nordberg, and P.-A. Hansson, “Soil Carbon Modelling in Salix Biomass Plantations: Variety Determines Carbon Sequestration and Climate Impacts,” *Forests*, vol. 12, no. 11, p. 1529, Nov. 2021, doi: 10.3390/f12111529.

[6] M. Weih and N.-E. Nordh, “Determinants of biomass production in hybrid willows and prediction of field performance from pot studies,” *Tree Physiology*, vol. 25, no. 9, pp. 1197–1206, Sep. 2005, doi: 10.1093/treephys/25.9.1197.

[7] TNO Biobased and Circular Technologies, “Phyllis2, database for (treated) biomass, algae, feedstocks for biogas production and biochar.” https://phyllis.nl/ (accessed Feb. 24, 2023).

[8] S. Chen, W. Liao, C. Liu, R. L. Kincaid, and and J. H. Harrison, “USE OF ANIMAL MANURE AS FEEDSTOCK FOR BIO-PRODUCTS,” in *Animal, Agricultural and Food Processing Wastes - IX*, American Society of Agricultural and Biological Engineers, 2003. doi: 10.13031/2013.15233.

[9] Z. Wen, “Hydrolysis of animal manure lignocellulosics for reducing sugar production,” *Bioresource Technology*, vol. 91, no. 1, pp. 31–39, Jan. 2004, doi: 10.1016/S0960-8524(03)00166-4.

[10] W. E. Jokela, J. P. Tilley, and D. S. Ross, “Manure Nutrient Content on Vermont Dairy Farms: Long‐Term Trends and Relationships,” *Communications in Soil Science and Plant Analysis*, vol. 41, no. 5, pp. 623–637, Mar. 2010, doi: 10.1080/00103620903531193.

[11] B. W. Thomas, X. Li, V. Nelson, and X. Hao, “Anaerobically Digested Cattle Manure Supplied More Nitrogen with Less Phosphorus Accumulation than Undigested Manure,” *Agronomy Journal*, vol. 109, no. 3, pp. 836–844, 2017, doi: 10.2134/agronj2016.12.0719.

[12] E. Kemppainen, “Nutrient content and fertilizer value of livestock manure with special reference to cow manure,” 1989, Accessed: Feb. 24, 2023. [Online]. Available: https://jukuri.luke.fi/handle/10024/441097

[13] M. Dougherty, D. H. Vaughan, G. K. Evanylo, E. R. Collins, Jr., and A. H. AbdelGadir, “Nitrogen Values of Liquid Dairy Manure and Dry Broiler Litter as Affected by Preservation Treatment,” *Applied Engineering in Agriculture*, vol. 25, no. 3, pp. 363–371, 2009, doi: 10.13031/2013.26887.
